# Alpha and gamma oscillation amplitudes synergistically predict the perception of forthcoming nociceptive stimuli

**DOI:** 10.1002/hbm.23048

**Published:** 2015-11-02

**Authors:** Yiheng Tu, Zhiguo Zhang, Ao Tan, Weiwei Peng, Yeung Sam Hung, Massieh Moayedi, Gian Domenico Iannetti, Li Hu

**Affiliations:** ^1^ Key Laboratory of Cognition and Personality (Ministry of Education) and Faculty of Psychology Southwest University Chongqing China; ^2^ Department of Electrical and Electronic Engineering the University of Hong Kong Hong Kong; ^3^ School of Chemical and Biomedical Engineering & School of Electrical and Electronic Engineering Nanyang Technological University Singapore; ^4^ School of Mobile Information Engineering Sun Yat‐Sen University Zhuhai China; ^5^ Department of Neuroscience, Physiology and Pharmacology University College London United Kingdom

**Keywords:** default mode networks, prestimulus alpha oscillations, prestimulus gamma oscillations, sensorimotor networks, sensory perception

## Abstract

Ongoing fluctuations of intrinsic cortical networks determine the dynamic state of the brain, and influence the perception of forthcoming sensory inputs. The functional state of these networks is defined by the amplitude and phase of ongoing oscillations of neuronal populations at different frequencies. The contribution of functionally different cortical networks has yet to be elucidated, and only a clear dependence of sensory perception on prestimulus alpha oscillations has been clearly identified. Here, we combined electroencephalography (EEG) and functional magnetic resonance imaging (fMRI) in a large sample of healthy participants to investigate how ongoing fluctuations in the activity of different cortical networks affect the perception of subsequent nociceptive stimuli. We observed that prestimulus EEG oscillations in the alpha (at bilateral central regions) and gamma (at parietal regions) bands negatively modulated the perception of subsequent stimuli. Combining information about alpha and gamma oscillations predicted subsequent perception significantly more accurately than either measure alone. In a parallel experiment, we found that prestimulus fMRI activity also modulated the perception of subsequent stimuli: perceptual ratings were higher when the BOLD signal was higher in nodes of the sensorimotor network and lower in nodes of the default mode network. Similar to what observed in the EEG data, prediction accuracy was improved when the amplitude of prestimulus BOLD signals in both networks was combined. These findings provide a comprehensive physiological basis to the idea that dynamic changes in brain state determine forthcoming behavioral outcomes. *Hum Brain Mapp 37:501–514, 2016*. © **2015 Wiley Periodicals, Inc.**

## INTRODUCTION

The Cartesian model of perception, a series of labeled lines resulting in the faithful encoding of stimulus features in the brain, has clearly been refuted. Instead, the dynamic state of the brain can dramatically modulate the perceptual outcome of a forthcoming stimulus [Engel et al., 2001]. Indeed, the fluctuations between brain states are characterized by the intrinsic dynamics of thalamocortical and corticocortical networks, which continuously modulate the neural processing of forthcoming sensory events [Fries, 2005; Keil et al., 2012; Lange et al., 2012]. Coherent fluctuations in activity of specific neuroanatomical systems define a number of cortical networks with different functional significance [Damoiseaux et al., 2006]. The levels of activity in these cortical networks can be correlated or anticorrelated, and can change independently in response to sensory stimulation or a cognitive task. At rest, ongoing fluctuations in these networks, which together define the dynamic state of the brain, determine the readiness of the system to respond to an external stimulus [Boly et al., 2007; Gilbert and Sigman, 2007].

The functional state of these distributed cortical networks is indexed by ongoing neuronal oscillations at different frequency bands (e.g., alpha: 8–14 Hz; beta: 14–30 Hz; gamma: 30–100 Hz), effectively measured by electroencephalography (EEG) or magnetoencephalography (MEG) [Hanslmayr et al., 2007]. Both power and phase of alpha and low‐beta oscillations in occipital areas have been demonstrated to influence neural responses elicited by subsequent visual stimuli, as well as their perceptual outcome [Busch et al., 2009; Hanslmayr et al., 2013; Mathewson et al., 2009; Van Dijk et al., 2008]. Similarly, the same oscillations in somatosensory areas modulate the responses elicited by tactile and nociceptive stimuli [Anderson and Ding, 2011; Babiloni et al., 2006a; Linkenkaer‐Hansen et al., 2004; Zhang and Ding, 2010]. Given that low‐power prestimulus alpha increases both the perception and the neural responses elicited by subsequent stimuli, alpha oscillations have been hypothesized to play an active role for the inhibitory control of their respective sensory cortices [Klimesch et al., 2007]. Gamma oscillations, which are important for long‐range communication between distributed neuronal ensembles [Fries, 2005], are another important candidate that may influence the perception of a forthcoming sensory stimulus, especially given that they are mechanistically important in several aspects of cognition, including attention‐dependent input selection and memory formation [Fries, 2009; Fries et al., 2007]. However, a link between prestimulus gamma and subsequent perceptual behavior has yet to be demonstrated. Moreover, since alpha and gamma oscillations are functionally different, we speculated that prestimulus alpha and gamma oscillations may have distinct predictive abilities, i.e., they might reflect brain states that can differentially predict the perception of subsequent stimuli.

Here, we tested these hypotheses by recording the brain activity from two large and independent samples of human subjects, using high‐density EEG (*n* = 96) and functional magnetic resonance imaging (fMRI, *n* = 32), respectively. First, we investigated the possible effects of the functional state of distributed cortical networks, as indexed by prestimulus EEG oscillations, on the psychophysical and neural responses elicited by a subsequent somatosensory stimulus. Second, we tested whether these modulatory effects are additive or multiplicative, by exploring whether they depend on the intensity of the incoming stimulus. Third, we formally tested the independence of the modulatory effects of prestimulus EEG oscillations, by quantifying their differential predictive values using a pattern recognition approach. Finally, we used fMRI to identify the brain areas whose functional state demonstrated an ability to predict perception akin to that of the alpha and gamma EEG oscillations by exploring the effect of prestimulus blood‐oxygen‐level‐dependent (BOLD) signal on the psychophysical and neural responses elicited by a subsequent stimulus.

## MATERIALS AND METHODS

### EEG Experiment

#### Participants

EEG data were collected from 96 healthy volunteers (51 females) aged 21.6 ± 1.7 years (mean ± SD, range = 17–25 years). All volunteers gave their written informed consent and were paid for their participation. The local ethics committee approved the experimental procedures. A different analysis of the same dataset was published [Hu et al., 2014].

#### Experimental design

Nociceptive‐specific radiant‐heat stimuli were generated by an infrared neodymium yttrium aluminum perovskite (Nd:YAP) laser with a wavelength of 1.34 μm (Electronical Engineering). These laser pulses selectively activate Aδ and C nociceptive terminals located within the epidermis, without coactivating Aβ fibers located in the dermis [Baumgärtner et al., 2005]. The laser beam was transmitted via an optic fiber and its diameter was set at approximately 7 mm (∼38 mm^2^) by focusing lenses. Laser pulses were directed on a square area (5 × 5 cm^2^) centered on the dorsum of the left hand. The pulse duration was 4 ms, and four stimulation intensities were used (E1: 2.5 J; E2: 3 J; E3: 3.5 J; E4: 4 J). After each stimulus, the target of the laser beam was shifted by approximately 1 cm in a random direction to avoid nociceptor fatigue or sensitization.

We delivered 10 laser pulses at each of the four stimulus intensities (E1–E4), for a total of 40 pulses, using a random and variable interstimulus interval between 10 and 15 s (rectangular distribution). The order of stimulus intensities was pseudorandomized. Three to six seconds after each stimulus, subjects were instructed to rate the intensity of the painful sensation elicited by the laser pulse, using a visual analog scale (VAS) ranging from 0 (corresponding to “no pain”) to 10 (corresponding to “pain as bad as it could be”) [Jensen and Karoly, 1992]. The perceived pain intensity at different stimulus energies was compared using a one‐way repeated‐measures analysis of variance (ANOVA). When the main effect was significant, post‐hoc Tukey's pairwise comparisons were performed.

#### EEG recording

Subjects were seated in a comfortable chair in a silent and temperature‐controlled room. They wore protective goggles and were asked to focus their attention on the stimuli and relax their muscles. EEG data were recorded using 64 Ag–AgCl scalp electrodes placed according to the International 10‐20 system (Brain Products GmbH; Munich, Germany; band pass: 0.01–100 Hz; sampling rate: 1,000 Hz), using the nose as reference. Electrode impedances were kept below 10 kΩ. Electro‐oculographic (EOG) signals were simultaneously recorded using surface electrodes to monitor ocular movements and eye blinks.

#### EEG data analysis

##### EEG data preprocessing and time domain analysis

EEG data were preprocessed using EEGLAB [Delorme and Makeig, 2004]. Continuous EEG data were bandpass filtered between 1 and 100 Hz, and segmented into epochs using a time window of 1,500 ms (−500 to 1000 ms relative to stimulus onset). EEG trials were baseline corrected using the prestimulus interval. Trials contaminated by eye blinks and movements were corrected using an independent component analysis algorithm [Delorme and Makeig, 2004]. In all datasets, removed independent components showed a large EOG channel contribution and a frontal scalp distribution. Finally, EEG trials with amplitudes exceeding ±100 µV (i.e., likely to be contaminated by artifacts) were excluded. For each subject, trials collected at each level of stimulus energy were averaged together, time‐locked to stimulus onset. This procedure yielded four average waveforms. Peak latencies and amplitudes of N2 and P2 waves, which were defined as the most negative and positive deflections between 150 and 500 ms after stimulus onset, were measured at Cz for each subject and stimulus intensity. N2 and P2 peak latencies and amplitudes at different stimulus intensities were respectively compared using a one‐way repeated‐measures ANOVA. When the main effect was significant, post‐hoc Tukey's pairwise comparisons were performed.

##### Time‐frequency analysis

Time‐frequency distributions of EEG trials were obtained using a windowed Fourier transform (WFT) with a fixed 200 ms Hanning window [Zhang et al., 2012]. This WFT yielded, for each EEG trial, a complex time‐frequency estimate *F*(*t*,*f*) at each time‐frequency point (*t*,*f*), extending from −500 to 1,000 ms (in steps of 1 ms) in the time domain, and from 1 to 100 Hz (in steps of 1 Hz) in the frequency domain. The resulting spectrogram, *P*(*t*,*f*) = ‖*F*(*t*,*f*)‖^2^, representing the signal power as a joint function of time and frequency at each time‐frequency point, contained brain responses both phase‐locked (event‐related potentials) and non‐phase‐locked (event‐related synchronization and desynchronization) to laser stimulation [Mouraux and Iannetti, 2008]. Since the focus of this study was to explore the influence of prestimulus EEG activity on both subjective pain intensity and poststimulus EEG responses, no baseline correction was performed on the time frequency distributions. Indeed, any time‐frequency baseline correction would unavoidably mix the variability of prestimulus and poststimulus EEG activities [Hu et al., 2014].

##### Partial least squares (PLS) analysis

For each subject, both EEG spectrograms and subjective pain intensities were normalized within each stimulus energy, by subtracting their respective means and dividing their standard deviations, to minimize the influence of stimulus energy on the assessment of their trial‐to‐trial relationship. For each subject and each electrode, the relationship between normalized EEG spectrogram and normalized subjective intensity of pain was described using a multivariate linear regression (MVLR) model [Hu et al., 2014]. The model coefficients, *α_t_*_,__*f*_, which captured the importance of EEG spectrogram at each time‐frequency point in the prediction of the intensity of pain, were estimated using a PLS analysis (please refer to Hu et al. [2014] for technical details).

To assess the significance of the relationship between the magnitude of time‐frequency EEG activity and the intensity of pain, a point‐by‐point one‐sample *t*‐test against zero, combined with nonparametric permutation testing [Maris and Oostenveld, 2007], was performed, separately for each electrode, on the estimated MVLR model coefficients *α_t_*_,__*f*_ to define significant time‐frequency clusters at each EEG electrode [Zhang et al., 2012]. Specifically, MVLR model coefficient *α_t_*_,__*f*_ at each time‐frequency point (*t*,*f*) was compared against zero using a one‐sample *t*‐test, yielding a time‐frequency map of *t* values. To account for the multiple‐comparison problem in the point‐by‐point statistical test, significant time‐frequency points (*p* < 0.05) were categorized in clusters based on their time‐frequency adjacency (cluster‐level statistical analysis). Only clusters composed of >20 adjacent significant time‐frequency points were considered, and only the largest cluster in the gamma range (≥30 Hz) was selected in the prestimulus and poststimulus intervals, to control for false‐positive observations. The cluster‐level statistics ( $\Sigma_{T}$) were defined by calculating the sum of the *t* values of all time‐frequency points within a cluster. For each subject, we randomly permutated 1000 times the subjective intensity of pain at each stimulus intensity to build a permuted MVLR model, and estimated the corresponding MVLR model coefficients. In each permutation (*m*‐th), the same one‐sample *t*‐test was performed on the permuted MVLR model coefficients at each time‐frequency point within the predefined clusters, which yielded a cluster‐level statistic $\sum_{T}^{*}\left(m\right)$. Permutation distributions $D(\Sigma_{T})$ of the cluster‐level *t*‐statistics were obtained from all $\sum_{T}^{*}\left(m\right)$, and the two‐tailed *p*‐value *p*_T_ was obtained by locating the observed $\Sigma_{T}$ under the permutation distribution *D*(*E*_T_) for each cluster [Benjamini and Hochberg, 1995].

To evaluate the strength of prestimulus effects across the scalp, we first modeled the relationship between EEG spectrograms within the identified time‐frequency clusters (i.e., prestimulus alpha oscillations: “Pre‐ABO,” and prestimulus gamma oscillations: “Pre‐GBO”; see Results section for details) from all electrodes and subjective intensity of pain. Model coefficients were then extracted and averaged across all time‐frequency points within each cluster for each electrode, resulting in the scalp topographies of model coefficients. In addition, to assess the possible influence of the stimulus energy on the relationship between EEG spectrogram and the intensity of pain within the defined prestimulus clusters, we performed a separate PLS analysis for each stimulus intensity (E1–E4). Within each prestimulus cluster, MVLR model coefficients *α_t_*_,__*f*_ at different stimulus intensities were compared using a one‐way repeated‐measures ANOVA. When the main effect was significant, post‐hoc Tukey's pairwise comparisons were performed.

##### Effects of prestimulus EEG activities

To assess the influence of each prestimulus feature on the perceived intensity of the subsequent somatosensory stimulus and the corresponding neural responses, single trials of each subject were sorted in ascending order according to the mean spectral power within the “Pre‐ABO” or “Pre‐GBO” time‐frequency clusters (measured from C4‐nose; the effect in other electrodes is reported in the Supporting Information). The bottom half of trials, reflecting the low “Pre‐ABO” or “Pre‐GBO,” and the top half of trials, reflecting the high “Pre‐ABO” or “Pre‐GBO,” as well as their corresponding pain intensities, were averaged. This procedure yielded two average waveforms of laser‐evoked potentials (LEPs) and two average values of intensity of pain for each subject and cluster. For each prestimulus feature, peak latencies and amplitudes of N2 and P2 waves were measured from the LEP waveform (Cz‐nose) for each subject and each prestimulus power level (low and high). N2 and P2 peak latencies and amplitudes, as well as the intensity of pain, were compared using a two‐way repeated‐measures ANOVA, with stimulus intensity (four levels: E1–E4) and prestimulus power (two levels: low and high) as within‐subject factors. When the interaction was significant, post‐hoc Tukey's pairwise comparisons were performed.

Additionally, to assess the predictive effects of “Pre‐ABO” and “Pre‐GBO” on the whole timecourse of the stimulus‐evoked EEG responses, the same two‐way repeated‐measures ANOVA (with stimulus energy and prestimulus power as within‐subject factors) was performed for each time‐point of the average LEP waveforms (Cz‐nose; the effects at all electrodes were reported in the Supporting Information). The significance of this analysis was assessed with a cluster‐based permutation testing, which was conceptually identical to the statistical approach for assessing the significance of the relationship between EEG spectrogram and subjective intensity of pain (described in the section titled “Partial least squares (PLS) analysis”). Significant time points (*p* < 0.05) in LEP waveforms were categorized in clusters based on their temporal adjacency.

##### Independence of prestimulus EEG features

To assess the physiological dependence between the two prestimulus features (“Pre‐ABO” and “Pre‐GBO”), we tested whether the linear, additive combination of these features (“Pre‐ABO + Pre‐GBO”) would significantly improve the prediction accuracy of the intensity of pain, compared to either prestimulus feature alone. The “Pre‐ABO” and “Pre‐GBO” powers were measured at C4 from their corresponding time‐frequency clusters for each trial, and the intensity of pain was normalized by subtracting the mean and dividing the standard deviation at each level of stimulus intensity to eliminate the systematic influence of stimulus intensity on the intensity of pain. Trials with normalized pain intensity lower and higher than 0 were respectively defined as low‐ and high‐pain trials. Pain prediction was achieved using a support vector machine (SVM) classifier with leave‐one‐out cross‐validation based on three feature sets: “Pre‐ABO” power, “Pre‐GBO” power, and the combination of “Pre‐ABO” and “Pre‐GBO” powers (“Pre‐ABO + Pre‐GBO”) (see Huang et al. [2013] for technical details of pain prediction). Therefore, prediction accuracy was obtained for each subject and each feature set. One‐way repeated‐measures ANOVA was used to compare the prediction accuracies of the three different prestimulus feature sets. When the main effect was significant, post‐hoc Tukey's pairwise comparisons were performed.

### FMRI Experiment

#### Participants

FMRI data were collected from 32 healthy volunteers (20 females) aged 22.1 ± 2.0 years (mean ± SD, range = 19–24 years). All volunteers gave their written informed consent and were paid for their participation. The local ethics committee approved the experimental procedures.

#### Experimental design

We delivered 10 laser pulses at each of the four stimulus intensities (E1–E4), for a total of 40 pulses, using a random and variable interstimulus interval between 27 and 33 s (rectangular distribution). The order of stimulus intensities was pseudorandomized. Subjects were instructed to move a slider to rate the intensity of the painful sensation elicited by the laser pulse 15–18 s after each stimulus, using an electronic 0–10 VAS (the left anchor was “no pain” and the right anchor was “pain as bad as it could be”). At the end of each trial, the slider automatically returned to the midpoint (VAS = 5). The intensity of pain at different stimulus energies was compared using a one‐way repeated‐measures ANOVA. When the main effect was significant, post‐hoc Tukey's pairwise comparisons were performed.

#### FMRI recording

Functional MRI data were acquired using a Siemens 3.0 T Trio scanner with a standard head coil at the Key Laboratory of Cognition and Personality (Ministry of Education) of Southwest University (China). A whole‐brain gradient‐echo, echo‐planar‐imaging sequence was used for functional scanning with a repetition time (TR) of 1,500 ms (29 ms echo time, 25 5.0‐mm‐thick slices with 0.5 mm interslice gaps, 3 × 3 mm in‐plane resolution, field of view 192 × 192 mm, matrix 64 × 64; flip angle = 90°). A high‐resolution, T1‐weighted structural image (1 mm^3^ isotropic voxel MPRAGE) was acquired after functional imaging.

#### FMRI data analysis

##### FMRI data preprocessing

The fMRI data were preprocessed and analyzed using SPM8 (Wellcome Trust Center for Neuroimaging, London, UK). The first five volumes were discarded to allow for signal equilibration. Images were corrected for slice‐timing and head motion. The resulting images were normalized to the Montreal Neurological Institute (MNI) space [Ashburner and Friston, 2005], spatially smoothed using a Gaussian kernel of 8 mm full width at half maximum (FWHM = 8 mm), and temporally filtered using a high‐pass filter with 1/128 Hz cutoff frequency.

##### General linear model analysis

Single‐subject fMRI data were analyzed on a voxel‐by‐voxel basis, using a general linear model (GLM) approach [Frackowiak et al., 2004]. For each stimulus energy, the BOLD responses were modeled as a series of events (laser pulses) using a stick function, which was then convolved with a canonical hemodynamic response function (HRF) [Downar et al., 2003]. Group‐level statistical analyses were carried out using a random effects analysis with a one‐sample *t*‐test, as implemented in SPM8. The significance threshold was set as P_FWE_ < 0.05 at cluster level in the whole‐brain exploratory analyses [Bennett et al., 2009].

##### PLS analysis

Similar to the EEG data analysis, both whole‐brain BOLD signals and the intensity of pain at each stimulus energy were normalized by subtracting their respective means and dividing their standard deviations, to minimize the effect of stimulus intensity on the assessment of their trial‐to‐trial relationship for each subject. The relationship between normalized BOLD signal at stimulus onset (which, because of the time‐lag of the hemodynamic response, reflect prestimulus brain activity) and the intensity of pain was modeled using MVLR, and estimated using the PLS analysis. The significance of these model coefficients, which reflected the effect of prestimulus brain activity at each voxel in predicting subjective pain intensity, was assessed using a one‐sample *t*‐test against zero, combined with cluster‐based nonparametric permutation testing. This analysis yielded significant clusters of brain regions, within which the prestimulus brain activity was predictive of the subjective intensity of the pain elicited by the forthcoming stimulus [Nichols and Holmes, 2002].

##### Independence of prestimulus BOLD features

To statistically assess the physiological independence of prestimulus BOLD signals that positively and negatively modulated the intensity of pain (“Pos‐BOLD” and “Neg‐BOLD”), we tested whether combining both features could significantly improve the prediction accuracy of intensity of pain, as compared to either feature alone. The onset “Pos‐BOLD” and “Neg‐BOLD” signals were respectively measured from the voxels that positively and negatively modulated intensity of pain for each single trial, and the intensity of pain was normalized by subtracting the mean and dividing the standard deviation at each level of stimulus intensity to eliminate the systematic influence of stimulus intensity on the intensity of pain. Trials with normalized pain intensity lower and higher than 0 were respectively defined as low‐ and high‐pain trials. Pain prediction was achieved using the SVM classifier with leave‐one‐out cross‐validation based on three feature sets: onset “Pos‐BOLD” signal, onset “Neg‐BOLD” signal, and their combination (“Pos‐BOLD + Neg‐BOLD”). The prediction accuracy was obtained for each subject and each feature set. A one‐way repeated‐measures ANOVA was used to compare the prediction accuracy of the three different feature sets. When the main effect was significant, post‐hoc Tukey's pairwise comparisons were performed.

## RESULTS

### EEG Results

#### Psychophysics

**Figure 1:**
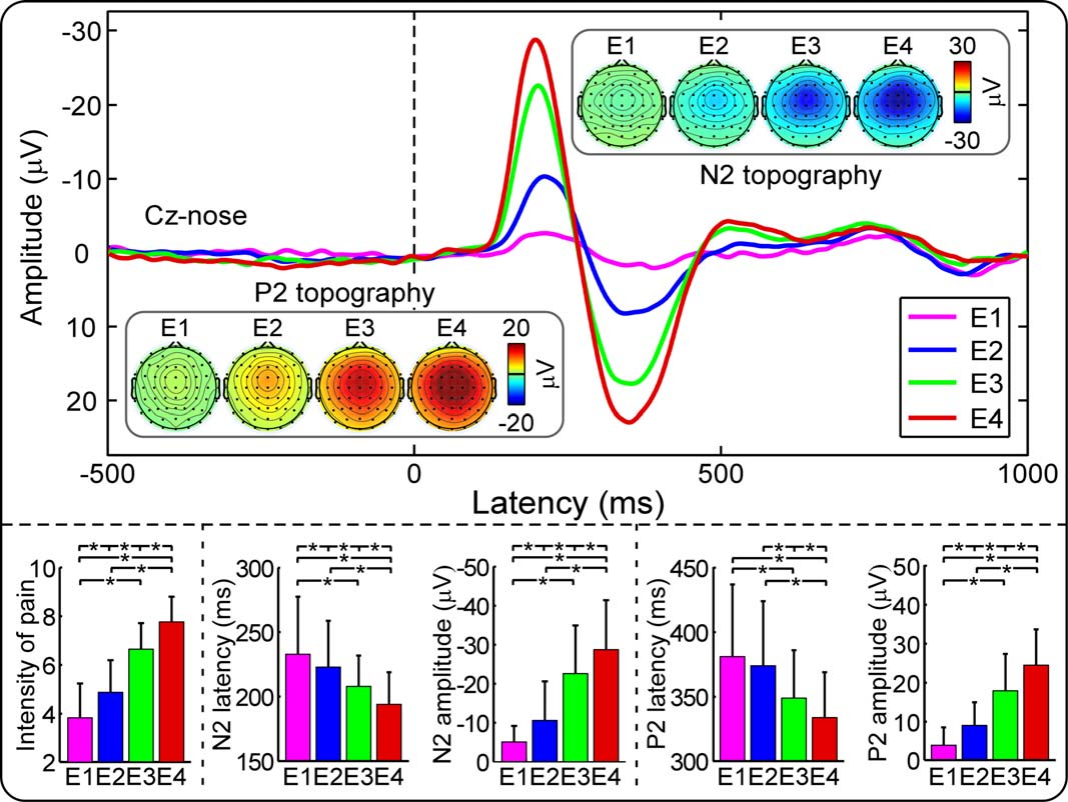
Laser‐evoked EEG responses in the time domain. Top panel: Group averages and scalp topographies of LEPs at different stimulus energies (E1: 2.5 J; E2: 3 J; E3: 3.5 J; E4: 4 J). LEPs were elicited by the stimulation of the left‐hand dorsum, and recorded from 64 channels, in 96 participants. Displayed waveforms were recorded from the vertex (Cz‐nose), and categorized accordingly to the stimulus intensity (colored waveforms). The scalp topographies of N2 and P2 waves are displayed at their peak latencies. Bottom panel: Subjective intensity of pain, N2 latency, N2 amplitude, P2 latency, and P2 amplitude at different stimulus intensities (E1–E4). Note how the stimulus intensity significantly and positively modulated intensity of pain, N2 and P2 amplitudes, but negatively modulated N2 and P2 latencies. [Color figure can be viewed in the online issue, which is available at http://wileyonlinelibrary.com.]

Nociceptive‐specific laser stimuli of four energies (E1–E4) elicited graded subjective intensities of pricking pain (E1: 3.8 ± 1.4; E2: 4.9 ± 1.3, E3: 6.6 ± 1.1, and E4: 7.7 ± 1.0). The one‐way repeated‐measures ANOVA revealed that the intensity of pain was significantly modulated by stimulus intensity (*F*_(3,93)_ = 527.2, *p* < 0.001; Fig. 1, bottom left). Post‐hoc Tukey's pairwise comparisons revealed that the intensity of pain was larger at higher stimulus energies (*p* < 0.05 for all comparisons).

#### Laser‐evoked EEG responses

The top panel of Figure 1 shows the group‐level waveforms of the LEPs elicited at Cz by stimuli at four different energies (E1–E4), and the scalp topographies at the peak latencies of the N2 and P2 waves. The latency and amplitude of the N2 and P2 waveforms were significantly modulated by stimulus intensity (Table 1), with shorter latencies and larger amplitudes for higher stimulus energies (Fig. 1; statistics are summarized in Table 2).

**Table 1:** N2 latency, N2 amplitude, P2 latency, and P2 amplitude elicited by laser stimuli of different intensities (E1–E4)

Stimulus intensity	N2 latency (ms)	N2 amplitude (μV)	P2 latency (ms)	P2 amplitude (μV)
E1	233 ± 45	−4.30 ± 4.92	382 ± 55	3.38 ± 5.14
E2	223 ± 36	−10.35 ± 10.10	374 ± 50	8.45 ± 6.12
E3	208 ± 24	−22.33 ± 12.49	349 ± 37	17.95 ± 9.01
E4	194 ± 25	−28.12 ± 14.82	332 ± 37	23.65 ± 9.25

**Table 2:** One‐way repeated‐measures ANOVA to assess the modulation of stimulus intensity (E1–E4) on N2 latency, N2 amplitude, P2 latency, and P2 amplitude

	N2 latency		N2 amplitude		P2 latency		P2 amplitude	
	*p* value	*F* value	*p* value	*F* value	*p* value	*F* value	*p* value	*F* value
ANOVA	<0.001	22.97	<0.001	327.33	<0.001	19.75	<0.001	288.07
Post‐hoc pairwise comparisons								
E1 versus E2	=0.02		<0.001		=0.65		<0.001	
E1 versus E3	=0.001		<0.001		<0.001		<0.001	
E1 versus E4	<0.001		<0.001		<0.001		<0.001	
E2 versus E3	<0.001		<0.001		<0.001		<0.001	
E2 versus E4	<0.001		<0.001		<0.001		<0.001	
E3 versus E4	<0.001		<0.001		=0.02		<0.001	

#### Subjective perception is dependent on prestimulus time‐frequency features

**Figure 2:**
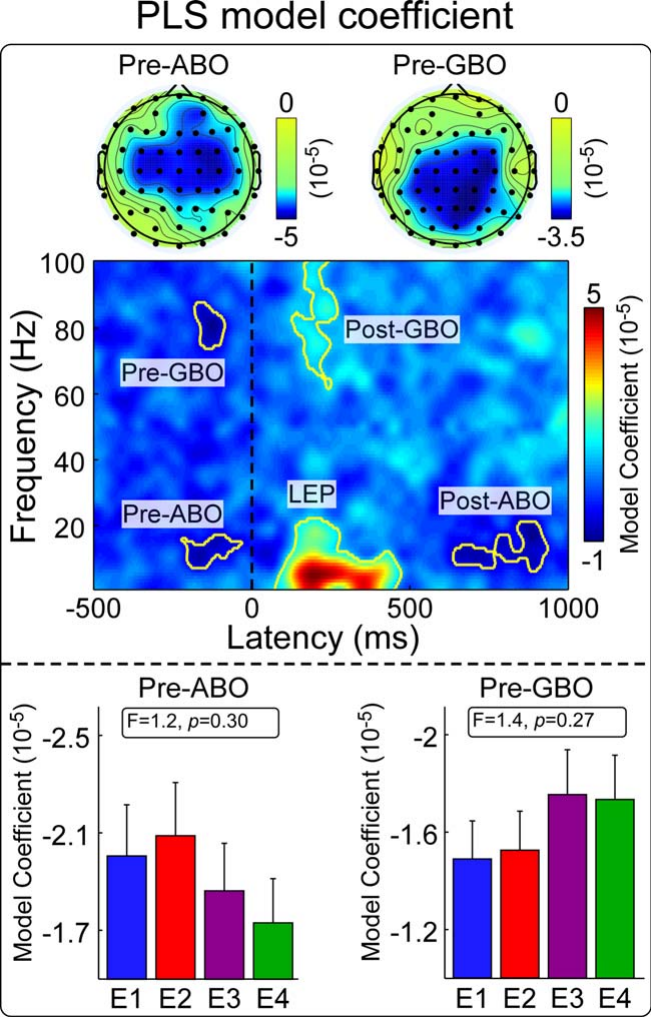
The relationship between laser‐elicited EEG spectrogram and the intensity of pain. Top panel: MVLR model coefficients indicating the relationship between EEG spectrogram and the intensity of pain. Five time‐frequency clusters of the EEG spectrogram (C4‐nose) were significantly modulated by the intensity of pain: “Pre‐ABO” (−221 to −31 ms, 8–15 Hz), “Pre‐GBO” (−180 to −85 ms, 74–87 Hz), “LEP” (74–470 ms, 1–22 Hz), “Post‐ABO” (637–935 ms, 8–20 Hz), and “Post‐GBO” (127–377 ms, 62–100 Hz). Scalp topographies of MVLR model coefficients within the “Pre‐ABO” and “Pre‐GBO” clusters are displayed in the top parts. Bottom panel: MVLR model coefficients within the “Pre‐ABO” and “Pre‐GBO” clusters at different stimulus intensities (E1–E4). Both parameters were not significantly modulated by stimulus intensity. [Color figure can be viewed in the online issue, which is available at http://wileyonlinelibrary.com.]

Two time‐frequency clusters in the prestimulus interval significantly modulated the perceived pain intensity (Fig. 2; C4‐nose): a cluster in the alpha frequency band (“Pre‐ABO”: −221 to −31 ms, 8–15 Hz; *p* < 0.001) and a cluster in the gamma frequency band (“Pre‐GBO”: −180 to −85 ms, 74–87 Hz; *p* = 0.001). The averaged MVLR model coefficients (mean ± SEM) within these two clusters were (−1.96 ± 2.24) × 10^−5^ and (−1.62 ± 1.89) × 10^−5^, respectively. This indicates that the magnitude of both prestimulus features negatively modulated the perceived intensity of a subsequent stimulus. The scalp topographies of these two prestimulus clusters were different: “Pre‐ABO” was located bilaterally over central regions, with a maximum at electrode C4, contralateral to stimulation site (i.e., a location roughly corresponding to the hand area in the primary sensorimotor cortex) [Valentini et al., 2012], while “Pre‐GBO” was distributed bilaterally over parietal regions, with a maximum around electrode CPz (Fig. 2).

Furthermore, in line with previous observations [Hu et al., 2014; Schulz et al., 2011], three significant time‐frequency clusters were observed in the poststimulus interval (electrode C4): the low‐frequency “LEP” (74–470 ms, 1–22 Hz; *p* < 0.001), the low‐frequency ABO (“Post‐ABO”: 637–935 ms, 8–20 Hz; *p* < 0.001), and the high‐frequency GBO (“Post‐GBO”: 127–377 ms, 62–100 Hz; *p* < 0.001). The average MVLR model coefficients (mean ± SEM) of these clusters were (5.72 ± 2.47) × 10^−5^, (−4.41 ± 2.77) × 10^−5^, and (3.82 ± 1.78) × 10^−5^, respectively, confirming that the subjective intensity of pain was positively correlated with the magnitudes of “LEP” and “Post‐GBO,” and negatively correlated with the magnitude of “Post‐ABO.” Similar time‐frequency clusters were also identified at other electrodes (Supporting Information, Fig. 1).

Importantly, the ability of the amplitude of alpha and gamma oscillations to modulate the subsequent perception was intensity‐independent, i.e., it was similar at different levels of stimulus intensity: MVLR model coefficients of both “Pre‐ABO” and “Pre‐GBO” were not significantly modulated by laser energy (E1–E4) (“Pre‐ABO”: *F*_(3,93)_ = 1.2, *p* = 0.30, “Pre‐GBO”: *F*_(3,93)_ = 1.4, *p* = 0.27; one‐way repeated‐measures ANOVA) (Fig. 2, bottom panels).

It should be noted that because the time‐frequency distributions of EEG trials were obtained using WFT with a 200 ms Hanning window, the estimates of prestimulus EEG time‐frequency data (−100 to 0 ms) were inevitably contributed by some poststimulus EEG data (0–100 ms), and vice versa. As a result, the model coefficients around stimulus onset (i.e., from −100 to 100 ms) necessarily represent the combination of both prestimulus (−100 to 0 ms) and poststimulus (0–100 ms) intervals, and should therefore be interpreted with caution.

#### Effects of prestimulus EEG activities on stimulus‐evoked neural responses

**Figure 3:**
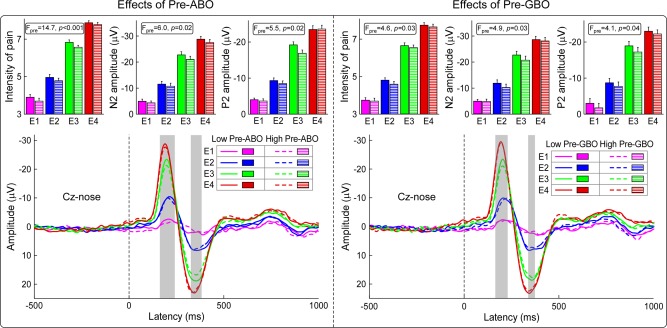
Influence of stimulus intensity and prestimulus EEG power on the subjective intensity of pain and LEP responses. Left panel: Influence of stimulus intensity (E1–E4) and “Pre‐ABO” power (low vs high) on the intensity of pain and LEP responses. The intensity of pain and the amplitude of the N2 and P2 LEP waves (Cz‐nose) were significantly increased with stimulus intensity, and decreased with “Pre‐ABO” power. LEP responses were significantly modulated by “Pre‐ABO” power within 159–235 ms and 348–398 ms (in grey). Right panel: Influence of stimulus intensity (E1–E4) and “Pre‐GBO” power (low vs high) on the intensity of pain and LEP responses (Cz‐nose). The intensity of pain and the amplitude of the N2 and P2 LEP waves (Cz‐nose) were significantly increased with stimulus intensity, and decreased with “Pre‐GBO” power. LEP responses were significantly modulated by “Pre‐GBO” power within 160–220 ms and 353–370 ms (in grey). [Color figure can be viewed in the online issue, which is available at http://wileyonlinelibrary.com.]

By median splitting the trials on the basis of the magnitude of the “Pre‐ABO” cluster, we assessed the effect of “Pre‐ABO” power and stimulus energy on subjective pain intensity, N2 amplitude, and P2 amplitude (Fig. 3, top‐left panel). All three responses were significantly modulated by both stimulus energy (pain: *F*_(3,93)_ = 435.8, *p* < 0.001; N2_amp_: *F*_(3,93)_ = 277.9, *p* < 0.001; P2_amp_: *F*_(3,93)_ = 159.8, *p* < 0.001) and “Pre‐ABO” power (pain: *F*_(1,95)_ = 14.7, *p* < 0.001; N2_amp_: *F*_(1,95)_ = 6.0, *p* = 0.02; P2_amp_: *F*_(1,95)_ = 5.5, *p* = 0.02): trials with smaller “Pre‐ABO” magnitudes were perceived as more painful and elicited N2 and P2 waves of larger amplitudes. There was no significant interaction between the two factors (pain: *F*_(3,93)_ = 0.6, *p* = 0.59; N2_amp_: *F*_(3,93)_ = 0.9, *p* = 0.44; P2_amp_: *F*_(3,93)_ = 2.2, *p* = 0.10), indicating that the effect of “Pre‐ABO” was independent of stimulus energy. In contrast, the latency of the N2 and P2 waves was significantly modulated by stimulus intensity (N2_lat_: *F*_(3,93)_ = 40.7, *p* < 0.001; P2_lat_: *F*_(3,93)_ = 54.7, *p* < 0.001), but not by “Pre‐ABO” power (N2_lat_: *F*_(1,95)_ = 1.0, *p* = 0.32; P2_lat_: *F*_(1,95)_ = 2.3, *p* = 0.10). The interaction between the two factors was not significant (N2_lat_: *F*_(3,93)_ = 2.3, *p* = 0.08; P2_lat_: *F*_(3,93)_ = 1.3, *p* = 0.29).

Similarly, we assessed the effect of “Pre‐GBO” power on subjective pain intensity, N2 amplitude, and P2 amplitude (Fig. 3, top‐right panel). All three responses were significantly modulated by both stimulus energy (pain: *F*_(3,93)_ = 499.2, *p* < 0.001; N2_amp_: *F*_(3,93)_ = 210.5, *p* < 0.001; P2_amp_: *F*_(3,93)_ = 155.4, *p* < 0.001) and “Pre‐GBO” power (pain: *F*_(1,95)_ = 4.6, *p* = 0.03; N2_amp_: *F*_(1,95)_ = 4.9, *p* = 0.03; P2_amp_: *F*_(1,95)_= 4.1, *p* = 0.04): trials with smaller “Pre‐GBO” magnitudes were perceived as more painful and elicited N2 and P2 waves of larger amplitudes. There was no significant interaction between the two factors (pain: *F*_(3,93)_ = 0.6, *p* = 0.60; N2_amp_: *F*_(3,93)_ = 0.8, *p* = 0.50; P2_amp_: *F*_(3,93)_ = 0.2, *p* = 0.89), indicating that the effect of “Pre‐GBO” was similar at all stimulus energies. Similar to what was observed for the “Pre‐ABO”, N2 and P2 latencies were significantly modulated by stimulus energy (N2_lat_: *F*_(3,93)_ = 34.0, *p* < 0.001; P2_lat_: *F*_(3,93)_ = 53.6, *p* < 0.001), but not by “Pre‐GBO” power (N2_lat_: *F*_(1,95)_ = 0.7, *p* = 0.59; P2_lat_: *F*_(1,95)_ = 1.6, *p* = 0.50). The interaction between the two factors was not significant (N2_lat_: *F*_(3,93)_ = 0.3, *p* = 0.85; P2_lat_: *F*_(3,93)_ = 0.6, *p* = 0.61).

Furthermore, the point‐by‐point analysis conducted on the entire LEP waveform at Cz revealed that LEP responses were significantly modulated by both “Pre‐ABO” power and “Pre‐GBO” power in two similar time intervals (“Pre‐ABO”: 159–235 and 348–398 ms; *p* = 0.002 and *p* = 0.005, respectively; “Pre‐GBO”: 165–220 and 353–370 ms; *p* = 0.004 and *p* = 0.007, respectively; Fig. 3, bottom panel). The same analysis conducted on the entire LEP waveforms across all electrodes showed that the influences of “Pre‐ABO” and “Pre‐GBO” powers on LEP responses (both N2 and P2 waves) were similarly maximal at central regions (see Supporting Information for details).

#### Different prestimulus EEG features predict perceptual outcome independently

**Figure 4:**
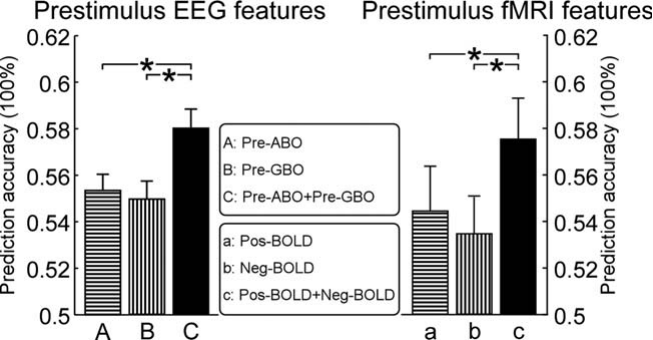
The performance to predict the subjective intensity of pain based on prestimulus features. For each level of stimulus energy, trials with normalized intensity of pain lower or higher than 0 were respectively defined as low‐ and high‐pain trials. Prediction accuracy to discriminate low‐ and high‐pain trials was estimated using SVM classifier and leave‐one‐out cross‐validation. Left panel: Using “Pre‐ABO,” “Pre‐GBO,” and “Pre‐ABO + Pre‐GBO” powers at C4‐nose, prediction accuracies to discriminate low‐ and high‐pain trials were 55.34 ± 0.69%, 54.97 ± 0.77%, and 58.02 ± 0.82%, respectively (*F*_(2,94)_ = 8.1, *p* < 0.001, one‐way repeated‐measures ANOVA). Right panel: Using onset “Pos‐BOLD,” “Neg‐BOLD,” and “Pos‐BOLD + Neg‐BOLD” signals, prediction accuracies to discriminate low‐ and high‐pain trials were 54.45 ± 1.87%, 53.44 ± 1.62%, and 57.50 ± 1.76%, respectively (*F*_(2,30)_ = 4.65, *p* = 0.013, one‐way repeated‐measures ANOVA). *p < 0.05.

To quantify the respective contribution of “Pre‐ABO” and “Pre‐GBO” power in determining the perceptual outcome of the subsequent stimulation, we calculated the prediction accuracy of perceived intensity based on each feature, or the combination of both. Prediction accuracies were as follows (mean ± SEM): 55.3 ± 0.7% (“Pre‐ABO”), 55.0 ± 0.8% (“Pre‐GBO”), and 58.0 ± 0.8% (“Pre‐ABO + Pre‐GBO”) (Fig. 4, left panel). These accuracy values were significantly different (*F*_(2,94)_ = 8.1, *p* < 0.001; one‐way repeated‐measures ANOVA). Post‐hoc Tukey's pairwise comparisons revealed that the prediction accuracy obtained based on the combined “Pre‐ABO + Pre‐GBO” power was significantly higher than the prediction accuracy based on either “Pre‐ABO” or “Pre‐GBO” power alone (*p* < 0.001 for both comparisons). This observation indicates that alpha and gamma oscillations likely reflect different cortical networks that influence the perception of subsequent somatosensory stimuli.

### Functional MRI Results

To circumvent the limitations posed by the spatial resolution of EEG source analysis, we used fMRI to sample the brain activity from an additional and independent sample of 32 healthy participants, during the same stimulation paradigm for the EEG experiments. We explored the effect of baseline fMRI signal on the psychophysical and neural responses elicited by a subsequent stimulus to identify the brain areas whose functional state showed a predictive ability similar to alpha and gamma EEG oscillations.

#### Psychophysics

Laser stimuli of the four energies elicited graded subjective pain intensities (E1: 2.9 ± 1.5; E2: 3.8 ± 1.7, E3: 5.7 ± 1.6, and E4: 6.9 ± 1.5). The one‐way repeated‐measures ANOVA revealed that the intensity of pain was significantly modulated by stimulus intensity (*F*_(3,29)_ = 163.51, *p* < 0.001). Post‐hoc Tukey's pairwise comparisons revealed that the intensity of pain was significantly larger at higher stimulus energies (*p* < 0.05 for all comparisons).

#### Laser‐evoked BOLD responses

**Figure 5:**
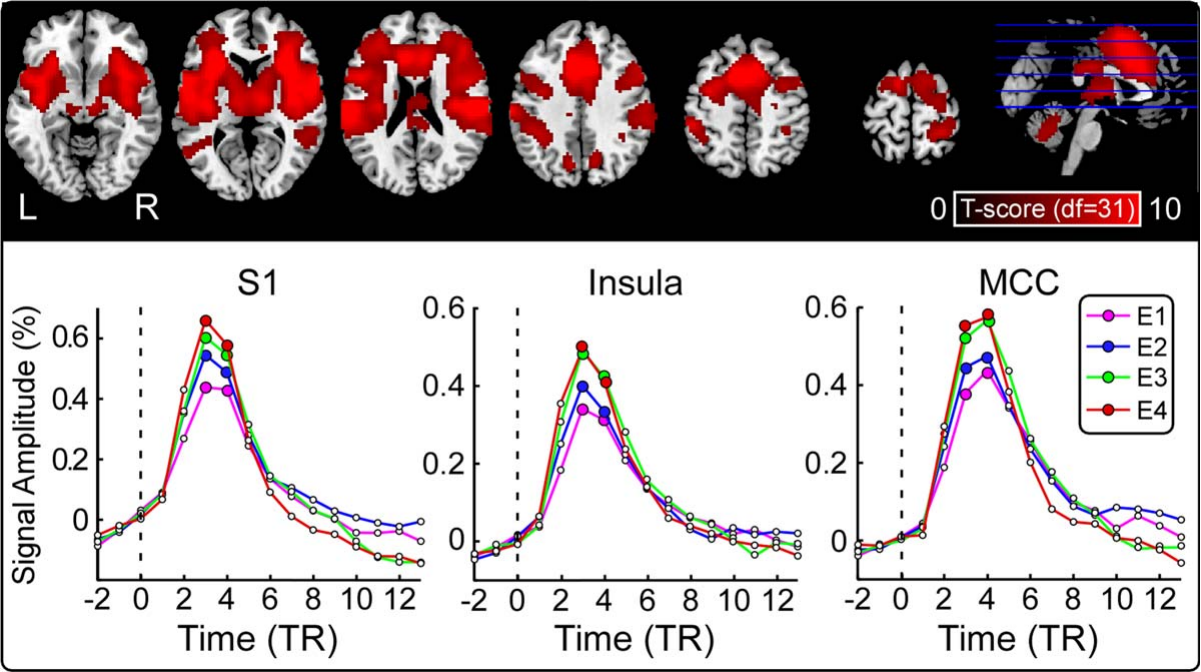
Laser‐evoked BOLD responses. Top panel: Brain regions activated by laser stimuli (cluster level: *P*_FWE_ < 0.05). Significant increases in BOLD signal to laser stimuli, across the four stimulus energies, are shown in red. L: left, R: right. Bottom panel: Average BOLD time courses in three representative regions (contralateral S1, contralateral insula, and MCC) at different stimulus energies (E1–E4). [Color figure can be viewed in the online issue, which is available at http://wileyonlinelibrary.com.]

Laser stimuli at each of the four energies elicited positive activations within a wide range of brain regions, including bilateral thalamus, bilateral primary somatosensory cortices (S1), bilateral secondary somatosensory cortices (S2), bilateral insula, and anterior and mid‐cingulate cortices (ACC and MCC) (cluster level: P_FWE_ < 0.05; Fig. 5, top panel). Group‐level BOLD time courses in some representative regions (contralateral S1, contralateral insula, and MCC) at different stimulus energies (E1–E4) are displayed in the bottom panel of Figure 5.

#### Subjective perception is dependent on prestimulus fMRI activity

**Figure 6:**
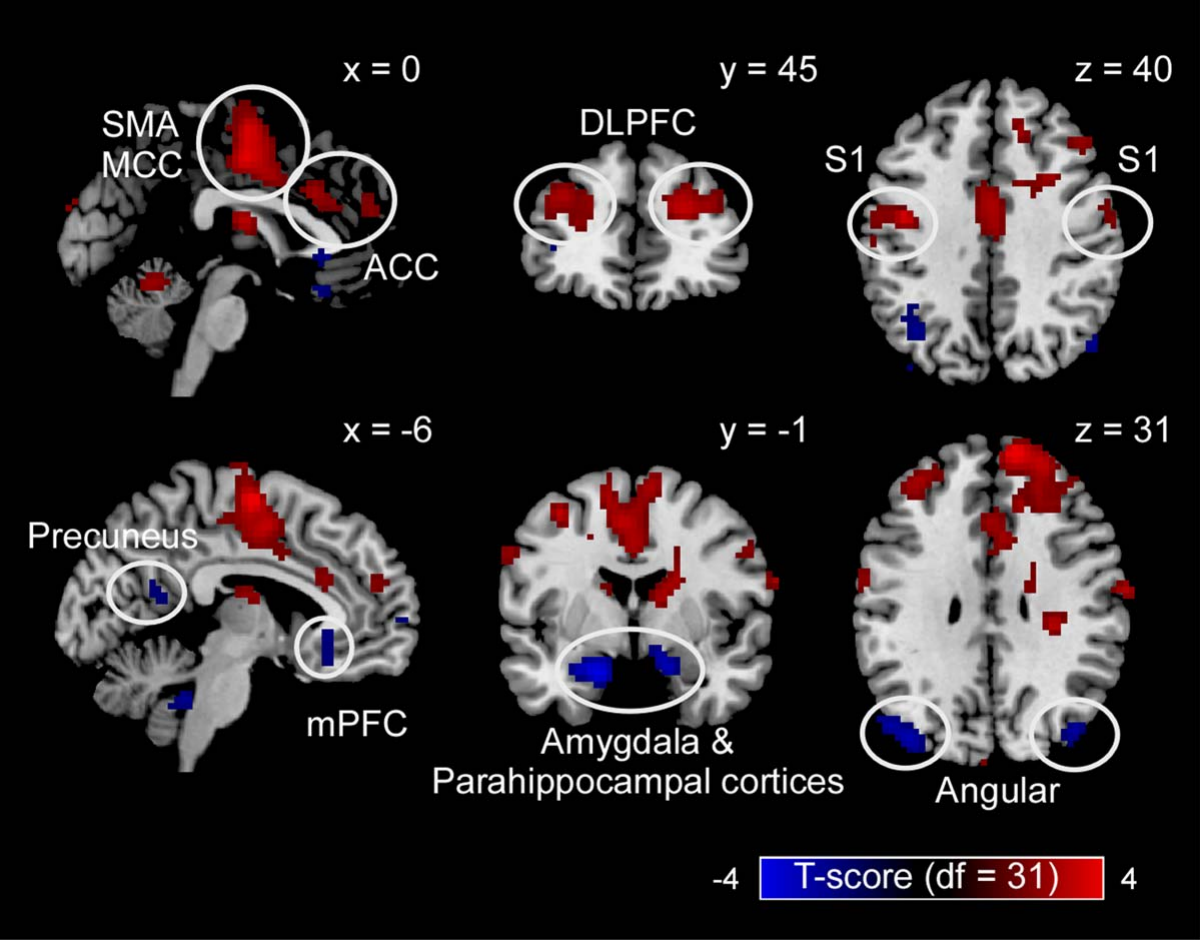
Brain regions whose prestimulus neural activity modulated subsequent pain perception. Prestimulus neural activity in bilateral primary somatosensory cortex (S1), supplementary motor area (SMA), anterior and mid‐cingulate cortices (ACC and MCC), and dorsolateral prefrontal cortex (DLPFC) positively modulated the perceived intensity of a subsequent painful stimulus. Prestimulus neural activity in medial prefrontal cortex (mPFC), bilateral precuneus, angular gyrus, and bilateral amygdala/parahippocampal cortices, negatively modulated the perceived intensity of a subsequent painful stimulus. Displayed voxels survived the voxel‐level threshold of *P*_uncorrected_ = 0.05, and the cluster‐level nonparametric permutation testing. [Color figure can be viewed in the online issue, which is available at http://wileyonlinelibrary.com.]

Prestimulus brain activity was measured using the fMRI signal sampled concomitantly to stimulus onset. Because of the intrinsic delay of the hemodynamic response [Jezzard et al., 2011], the fMRI signal sampled at stimulus onset (TR = 0) reflects the neural activity preceding the arrival of the sensory input to the nervous system. Neural activity in several brain regions showed the ability to significantly modulate the perceptual outcome of the subsequent stimuli, regardless of stimulus energy (Fig. 6, top panel). A positive prediction of subsequent pain perception was observed in bilateral S1, supplementary motor area (SMA), ACC, MCC, and dorsolateral prefrontal cortex (DLPFC)—which we collectively call as “Pos‐BOLD” regions hereafter. A negative prediction of subsequent pain perception was observed in medial prefrontal cortex (mPFC), bilateral precuneus, angular gyrus, and bilateral amygdala/parahippocampal cortices—which we collectively call as “Neg‐BOLD” regions hereafter.

#### Different features of the prestimulus fMRI activity predict perceptual outcome complementarily

To quantify the respective contribution of prestimulus activity in “Pos‐BOLD” and “Neg‐BOLD” regions in determining the perceptual outcome of the subsequent stimulation, we calculated the prediction accuracy of perceived intensity based on each region, and on their combination. Prediction accuracies (mean ± SEM) were as follows: 54.4 ± 1.9% (“Pos‐BOLD”), 53.4 ± 1.6% (“Neg‐BOLD”), and 57.5 ± 1.8% (“Pos‐BOLD + Neg‐BOLD”) (Fig. 4, right panel). These accuracy values were significantly different (*F*_(2,30)_ = 4.65, *p* = 0.013; one‐way repeated‐measures ANOVA). Importantly, post‐hoc Tukey's pairwise comparisons revealed that the prediction accuracy obtained based on onset “Pos‐BOLD + Neg‐BOLD” signal was significantly higher than that based on either onset “Pos‐BOLD” or “Neg‐BOLD” signal alone (*p* < 0.05 and *p* < 0.001, respectively). Similar to what observed with prestimulus alpha and gamma EEG oscillations, “Pos‐BOLD” and “Neg‐BOLD” regions have complementary predictive powers, suggesting that they reflect different cortical networks that are able to influence the perceptual outcome of subsequent stimulation.

## DISCUSSION

Characterizing how spontaneous fluctuations in the activity of distinct functional networks influence the perception of forthcoming events is important for understanding the mechanisms by which sensory stimuli are perceived. In this study, we collected EEG and fMRI data in two large samples of human participants. Both experiments provided converging evidence that the perceived intensity of a nociceptive somatosensory stimulus is clearly dependent on the state of brain immediately preceding the stimulus. We obtained two main findings.

First, two distinct electrophysiological features (identified using scalp EEG)—alpha oscillations at bilateral central regions and gamma oscillations at parietal regions—can predict both the intensity of perception and the brain responses elicited by a subsequent somatosensory stimulation. The predictive ability of these prestimulus EEG features was independent from the intensity of the incoming sensory stimulation, indicating an intracortical algebraic modulation, rather than a spinal presynaptic inhibition. Importantly, the information contained in prestimulus alpha and gamma oscillations act synergistically in predicting the subsequent perception, indicating that these two electrophysiological features likely reflect distinct functional features.

Second, using fMRI, we identified two distinct sets of brain areas whose level of baseline functional activity predicted the perception of subsequent stimuli in different directions. High baseline activity in S1, SMA, ACC, MCC, and DLPFC predicted higher perceived intensity, whereas low baseline activity in mPFC, precuneus, angular gyrus, amygdala, and parahippocampal cortices predicted lower perceived intensity. Similar to what was observed in the EEG experiment, combining the prestimulus fMRI signal from both positively and negatively modulating regions significantly improved the prediction of the subsequent perception. This finding confirms that the positively and negatively modulating areas reflect functionally independent resting‐state networks (RSNs) [Damoiseaux et al., 2006].

An interesting observation was the spatial congruence of the results obtained in the EEG and fMRI experiments. Indeed, the scalp distribution of prestimulus EEG oscillations in the alpha band was congruent with the spatial distribution of a subset of regions that showed a positive predictive value of pain intensity in fMRI, namely the bilateral S1. Therefore, prestimulus alpha oscillations may partly reflect the neural activity of the sensory‐motor RSN [Anderson and Ding, 2011; Damoiseaux et al., 2006; Haegens et al., 2011; Weisz et al., 2014; Zhang and Ding, 2010]. Similarly, the scalp distribution of prestimulus gamma oscillations was compatible with some neural assembles in default model network (DMN), i.e., a subset of brain areas that have a negative predictive value on the pain intensity evoked by the subsequent stimulation, namely the precuneus and the angular gyrus. Therefore, prestimulus gamma oscillations likely represent the electrophysiological correlate of at least part of the neural activity of the DMN.

### Prestimulus Alpha Oscillations and the Sensorimotor RSN

A clear cluster of prestimulus alpha oscillations (−221 to −31 ms, 8–15 Hz), located bilaterally around central electrodes and maximal at C4, predicted both the intensity of perception and the neural responses elicited by a subsequent nociceptive stimulus (Figs. 3 and 4). This finding is consistent with a number of studies [Babiloni et al., 2006a; Busch et al., 2009; Busch and VanRullen, 2010; Hanslmayr et al., 2007; Mathewson et al., 2009; Van Dijk et al., 2008; Zhang and Ding, 2010], showing that the magnitude of prestimulus oscillations in the alpha band influences the perceptual outcome of subsequent sensory stimuli. This modulatory effect seems to be dependent on the functional state of the primary sensory cortex pertinent to the modality of the forthcoming stimulus. For example, trials with reduced alpha power in the occipital region result in increased awareness of subsequent visual stimuli [Babiloni et al., 2006b; Hanslmayr et al., 2007; Van Dijk et al., 2008]. Similarly, prestimulus alpha oscillations in the sensorimotor cortex modulate the detectability of subsequent weak tactile stimuli [Anderson and Ding, 2011; Weisz et al., 2014; Zhang and Ding, 2010]. Considering their modality‐dependent scalp distributions, prestimulus alpha oscillations have been interpreted as a measure of altered excitability of neuronal ensembles in primary sensory cortices [Jensen and Mazaheri, 2010; Klimesch et al., 2007]. This hypothesis has been confirmed by several fMRI studies showing that intensity of perception in a given sensory modality is predicted by prestimulus increases of BOLD signal in the corresponding primary sensory cortices [Boly et al., 2007; Brodersen et al., 2012; Rahnev et al., 2012]. In line with these studies [Boly et al., 2007; Brodersen et al., 2012], we observed that prestimulus neural activity in bilateral S1 modulated the perceived intensity of a forthcoming somatosensory stimulus (Fig. 6). Interestingly, prestimulus alpha oscillations that predicted subsequent perception showed a scalp distribution with two maxima in the bilateral central regions (electrodes C3 and C4). Therefore, it is possible that the prestimulus alpha oscillations identified in the EEG experiments represent the electrophysiological counterpart of the BOLD activity detected in bilateral S1. Notably, high baseline activity in a number of other areas (SMA, ACC, MCC, and DLPFC) also predicted the perception toward a stronger intensity. However, the baseline activity of these areas might have not been reflected in the EEG datasets, either because neural activity in those areas did not translate into an EEG signal measurable at scalp level or because the electrophysiological counterpart of the baseline activity of these areas was too weak to reach significance at the stringent statistical threshold that we used [Nunez and Srinivasan, 2006].

### Prestimulus Gamma Oscillations and the DMN

Gamma oscillations modulate long‐range communication between distributed neuronal assembles, and thereby subserve a range of cognitive operations, including feature binding, multisensory integration, and attention‐dependent input selection [Fries et al., 2007; Herrmann et al., 2004; Jensen et al., 2007; Klimesch et al., 2007; Ward, 2003]. In contrast to alpha oscillations, the influence of the power of gamma oscillations on the perception of subsequent sensory stimuli has rarely been reported [Reinhart et al., 2011], which is likely explained by their low signal‐to‐noise ratio [Babiloni et al., 2006a; Busch et al., 2009]. The large sample size of our EEG dataset (96 participants) has allowed us to detect effects that could have been missed in studies conducted on smaller samples. Consequently, we were able to clearly show that prestimulus gamma oscillations in parietal regions (−180 to −85 ms, 74–87 Hz, maximal around CPz) can modulate both the perceived intensity and the neural responses elicited by a subsequent somatosensory stimulus (Figs. 3 and 4). The combination of prestimulus alpha and gamma oscillation magnitudes improved the prediction of subsequent pain perception (Fig. 4). This is an important finding as it indicates that spontaneous fluctuations of these two features complementarily predict subsequent perception. We speculate that the negative modulation exerted by prestimulus gamma oscillations detected using EEG and the prestimulus neural activity detected using fMRI in the DMN are partly functionally related, based on two lines of reasoning. First, congruent to the combination of prestimulus ABO and GBO, combining prestimulus fMRI signals from brain regions that either positively or negatively modulate pain perception had a synergistic effect on the accuracy in predicting subsequent pain perception (Fig. 6). Notably, not only did high baseline fMRI activity in the sensorimotor RSN exert a facilitatory effect upon perception of subsequent sensory stimuli, but so did low baseline fMRI activity in nodes of the DMN (including the mPFC, precuneus, angular gyrus, amygdala, and parahippocampal cortices; Fig. 6) [Boly et al., 2007; Brodersen et al., 2012; Ploner et al., 2010]. Considering the correspondence between the scalp topography of positively predicting prestimulus alpha oscillations and the anatomical location of positively predicting S1 areas, it is reasonable to hypothesize that prestimulus gamma oscillations observed in the EEG experiment could similarly reflect part of the areas showing a negative prediction in the fMRI experiment (Fig. 6). Indeed, the electrodes, where gamma oscillations negatively predicted subsequent stimulus perception, were clustered around the midline parietal region, with a maximum at CPz. This scalp distribution is consistent with the activity of some of the nodes of the DMN identified by fMRI (i.e., the precuneus and angular gyrus). Second, both prestimulus gamma oscillations and prestimulus fMRI signals in the DMN negatively predicted the perceived intensity of subsequent stimuli. Therefore, there seems to be converging evidence indicating that gamma oscillations over parietal regions could reflect the state of part of the DMN that has shown in the fMRI experiment to be able to negatively predict the perception outcome of subsequent stimulation.

Even though we took care of using similar experimental designs in the EEG and fMRI experiments, the link between the EEG and fMRI results is not straightforward. For example, the possible neural generators of prestimulus alpha and gamma oscillations are difficult to pin down. Future studies should confirm whether prestimulus alpha and gamma oscillations may partly reflect the neural activity of the sensory motor RSN and the DMN, respectively.

## CONCLUSION

In summary, our findings provide a more comprehensive physiological basis to the idea that dynamic changes in brain state determine forthcoming perceptual and neurophysiological responses in humans [Gilbert and Sigman, 2007]. Particularly, they provide novel electrophysiological evidence supporting the existence of concurrent and independent neuronal oscillations and brain networks with different functional significance [Boly et al., 2007; Brodersen et al., 2012; Ploner et al., 2010], whose activities jointly bias perception and neural responses elicited by subsequent somatosensory stimuli.

## Supporting information

Supporting Information
