# Supplementary material for: Alpha and gamma oscillation amplitudes synergistically predict the perception of forthcoming nociceptive stimuli
Source: Hum Brain Mapp. 2015 Nov 2;37(2):501–14. doi: 10.1002/hbm.23048 (PMC4843944; doi:10.1002/hbm.23048)
Supplement: Supplementary file 1 — Supporting Information [file HBM-37-501-s001.doc]

**Supplementary material** for “Alpha and gamma oscillation amplitudes synergistically predict the perception of forthcoming nociceptive stimuli”


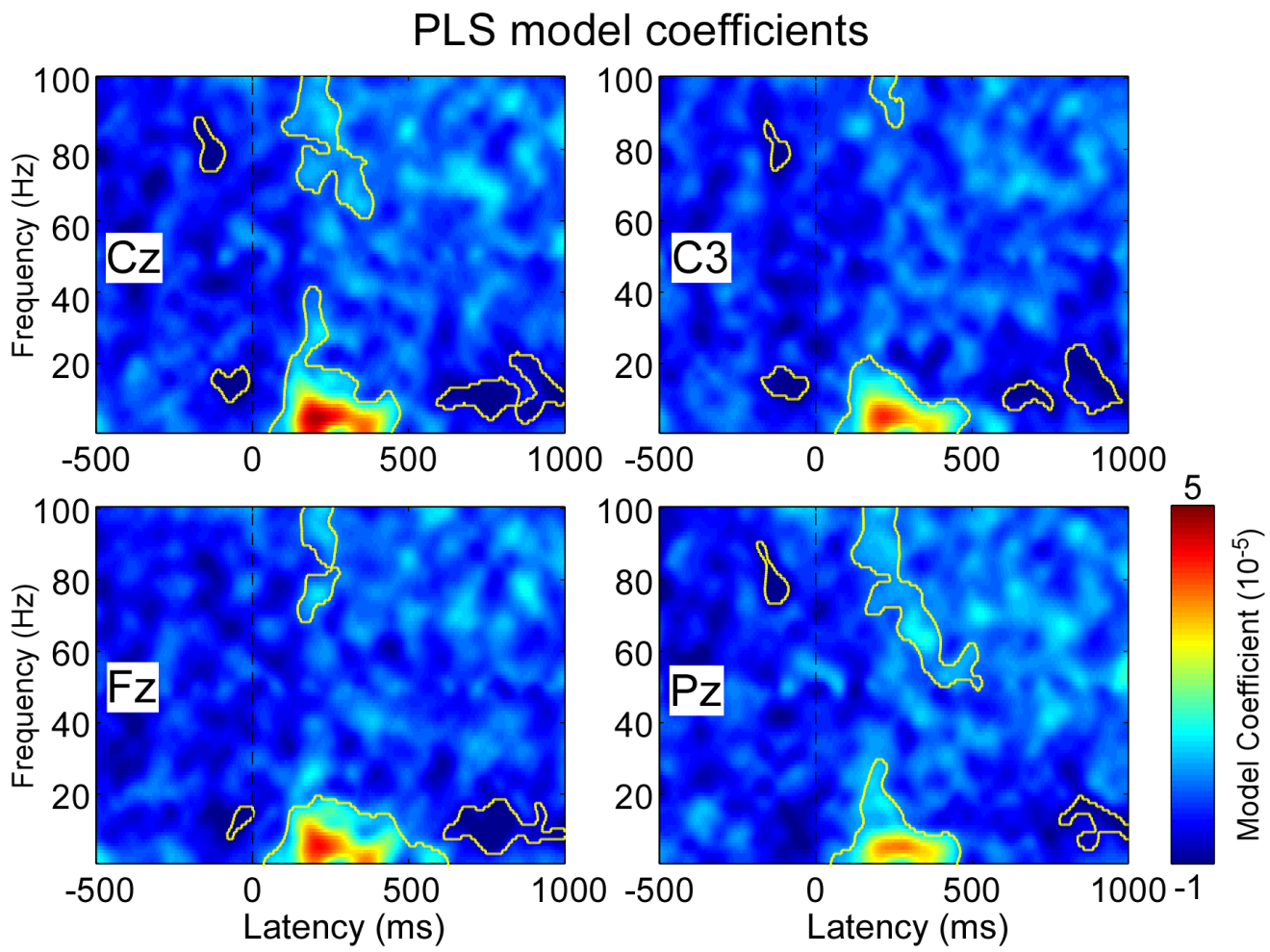


**Supplementary figure 1.** MVLR model coefficients indicating the relationship between the subjective intensity of pain and EEG spectrograms at Cz, C3, Fz, and Pz respectively (nose reference). Significant time-frequency clusters, marked in each plot, were identified using one-sample t-test combined with the nonparametric permutation testing. Note that clear ‘Pre-ABO’ was identified from Cz, C3, and Fz, and that clear ‘Pre-GBO’ was identified from Cz, C3, and Pz.

**
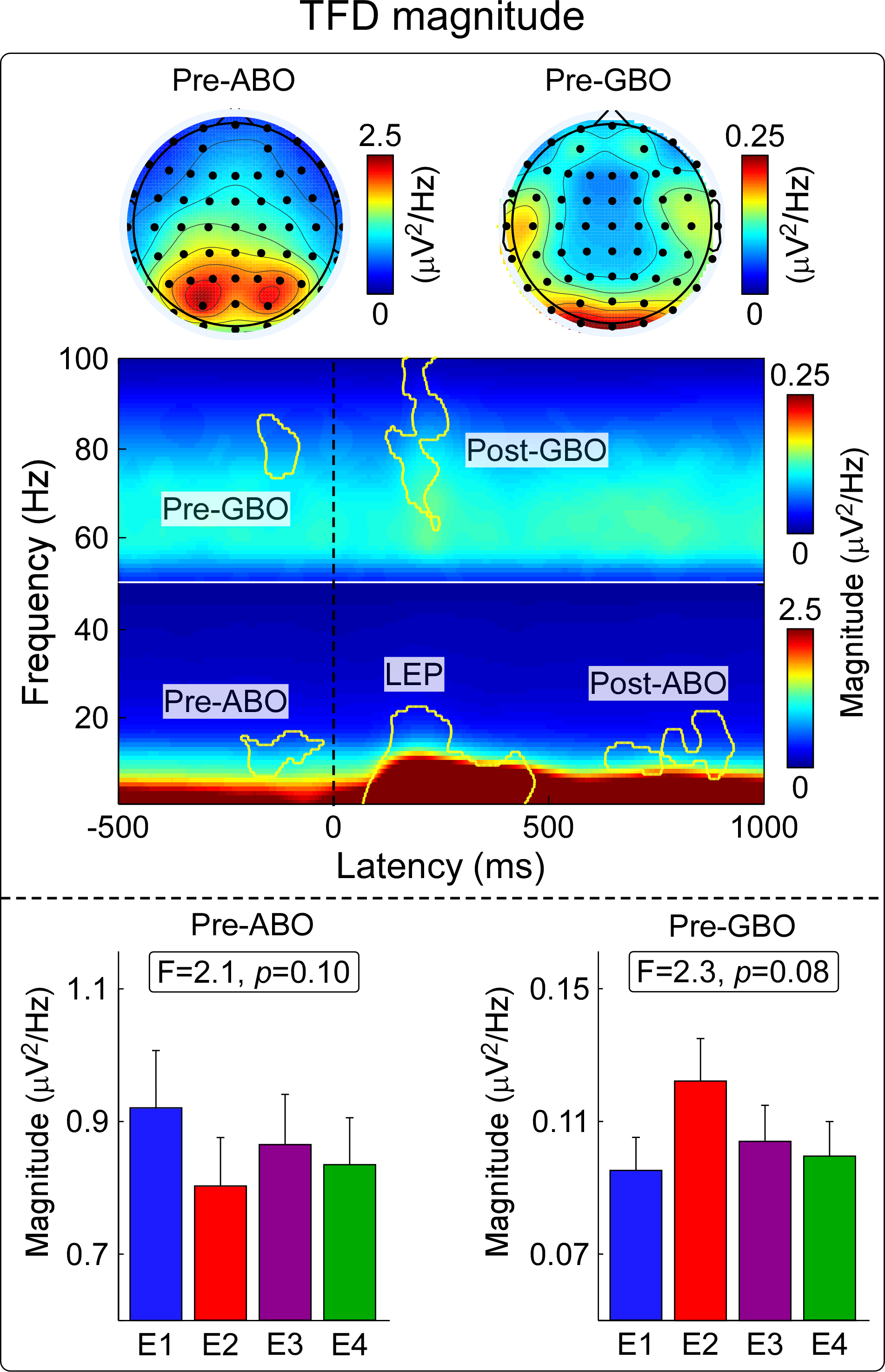
**

**Supplementary figure 2.** *Top panel:* Group-level average of the EEG spectrogram (C4-nose) and scalp topographies of 'Pre-ABO' power and 'Pre-GBO' power are displayed in the top panel. *Bottom panel:* 'Pre-ABO' power and 'Pre-GBO' power at different intensities (E1-E4). One-way repeated measures ANOVA showed both 'Pre-ABO' power and 'Pre-GBO' power were not significantly modulated by stimulus energy.


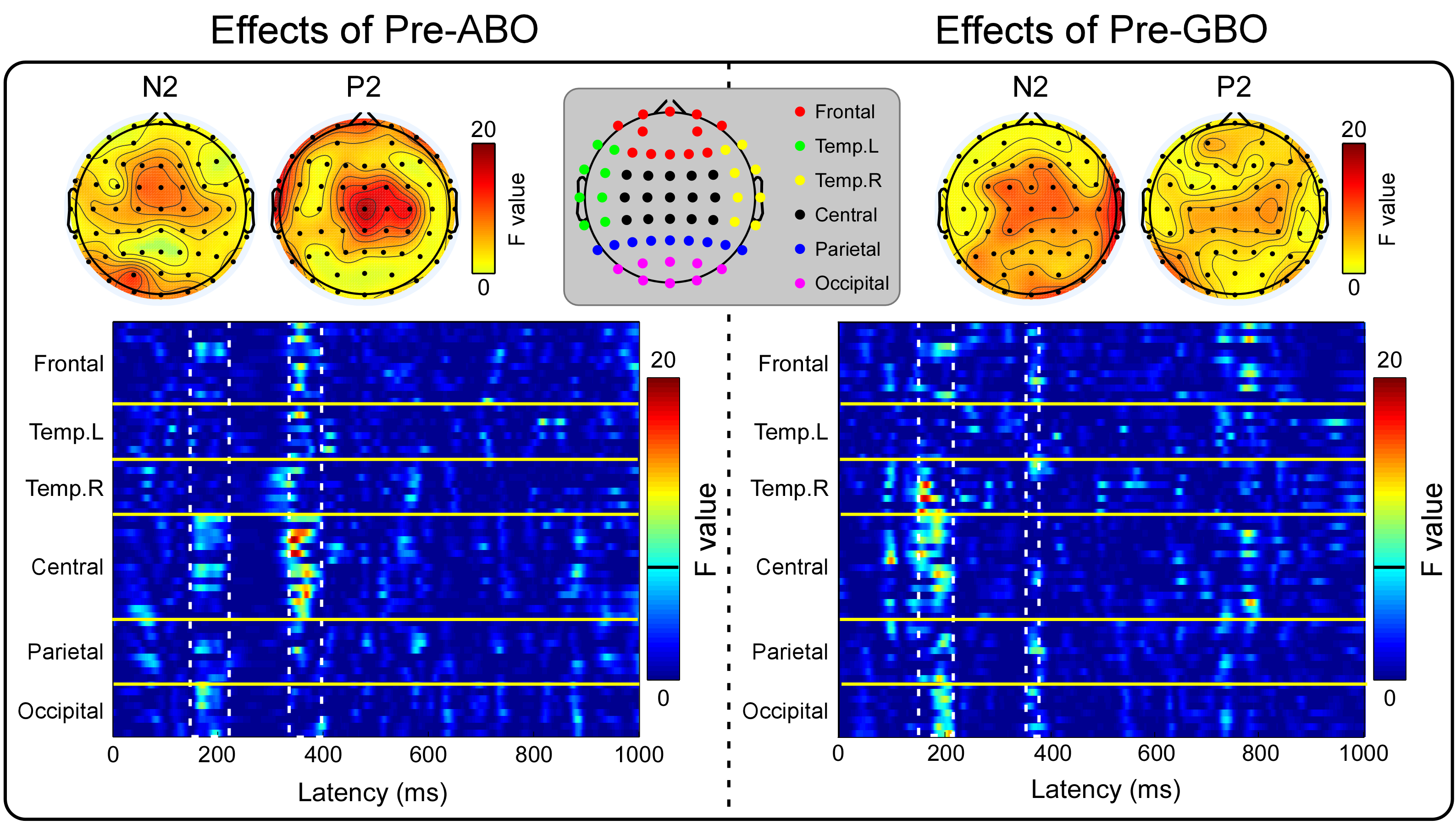


**Supplementary figure 3.** Influence of prestimulus EEG power on LEP responses across all electrodes. *Left panel*: Influence of 'Pre-ABO' power (low vs. high) on LEP responses. *Right panel*: Influence of 'Pre-GBO' power (low vs. high) on LEP responses. Scalp topographies of F values showed that the influences of ‘Pre-ABO’ and ‘Pre-GBO’ powers on LEP responses (both N2 and P2 waves) were similarly maximal at central regions.
